# Increased Cathepsin D Correlates with Clinical Parameters in Newly Diagnosed Type 2 Diabetes

**DOI:** 10.1155/2017/5286408

**Published:** 2017-12-10

**Authors:** Lei Liu, Baoxian Chen, Xudong Zhang, Lun Tan, Dao Wen Wang

**Affiliations:** ^1^Department of Internal Medicine, Tongji Hospital, Huazhong University of Science and Technology, Wuhan, China; ^2^Department of Medical Ultrasound, Tongji Hospital, Huazhong University of Science and Technology, Wuhan, China

## Abstract

**Background:**

Cathepsin D has been recently implicated in insulin resistance and cardiovascular disease. This study was designed to investigate the relationship between cathepsin D and newly diagnosed type 2 diabetes.

**Methods:**

Circulating cathepsin D levels and metabolic variables were measured in 98 cases and 98 controls. Myocardial performance index “Tei index” that reflects both left ventricular systolic and diastolic function was measured with Doppler echocardiography in cases.

**Results:**

Newly diagnosed type 2 diabetes demonstrated significantly higher circulating cathepsin D concentrations than controls (median level: 227 ng/ml versus 174 ng/ml, *P* < 0.01). In newly diagnosed type 2 diabetes, a significant correlation was found between cathepsin D levels and HOMA-IR (homeostatic model assessment of insulin resistance) (*r* = 0.25, *P* = 0.01). In contrast, no significant correlation was found between cathepsin D levels and clinical parameters in the control group (all *P* > 0.05). Interestingly, correlation analysis revealed a positive association between cathepsin D levels and Tei index in type 2 diabetes (*r* = 0.22, *P* = 0.03).

**Conclusions:**

Increased levels of circulating cathepsin D are closely linked with the presence of type 2 diabetes, and cathepsin D might serve as a novel biomarker for cardiac dysfunction in newly diagnosed type 2 diabetes.

## 1. Introduction

Emerging technologies have allowed the feasibility of acquiring high-throughput proteomic blood profiling from a blood specimen [1]. These techniques enable assessment of large amount of protein whether they have unanticipated roles as regulatory signals in various pathophysiological pathways. Furthermore, in addition to serving as potential biomarkers of disease, it may provide many additional insights regarding pathophysiological mechanisms. Although previous studies have documented that lots of protein biomarkers are used for diagnosis and management of cancers and other diseases [2, 3], studies on novel biologic predictors for insulin resistance and diabetes remain to be investigated. Given the prevalence of diabetes continues to increase in epidemic proportions and its complications remain major causes of morbidity and mortality [4–6], earlier identification of individuals at risk for diabetes is particularly important.

Recently, altered circulating cathepsin D levels have been described in two large community cohorts with prevalent insulin resistance by using proximity extension assay [7]. Extracellular matrix proteomics have also identified cathepsin D for high-risk atherosclerotic plaques [8]. Although the exact biological mechanism underlying the association between cathepsin D levels and insulin resistance and cardiovascular disease remains uncertain, it has attracted increasing interests. What is more, if these findings successfully confirmed in the clinic, this would open a new door to a possible pathogenesis of type 2 diabetes and cardiovascular disease. Cathepsin D, which plays an important role in maintaining tissue homeostasis and metabolism, is an aspartyl protease responsible for the degradation of intracellular and endocytosed proteins, representing one of the major endopeptidase activities in lysosomes [9]. Although cathepsin D has been implicated in acidic milieu of lysosomes with important consequences in regulation of apoptosis, it represents an important prognostic factor in a variety of cancers and is therefore considered to be a potential important molecule and influences cell signaling [10]. Previous studies have suggested that cathepsin D plays an important role in cholesterol trafficking and atherosclerosis [11, 12]. Cathepsin D has been proposed as a biomarker for nonalcoholic steatohepatitis [13]. Additionally, cathepsin D has also been linked to neurodegenerative disorders and in particular to Alzheimer's disease [14, 15]. Of note, cathepsin D has been implicated in apoptosis of myocardium, which presents extracellularly under pathological conditions. Clinical follow-up analyses showed that serum cathepsin D level in acute myocardial infarction patients was inversely related to cardiac dysfunction [16].

Despite the important roles of cathepsin D in many physiological and pathological conditions, whether its circulating levels associated with diabetes and clinical variable remain to be established. Accordingly, we set out to investigate circulating cathepsin D concentrations and their associations with indexes of insulin resistance and clinical variables in normal individuals and newly diagnosed type 2 diabetic patients.

## 2. Research Design and Methods

### 2.1. Study Design and Subjects

In order to examine the relationship between circulating cathepsin D concentrations and indexes of insulin resistance and various metabolic variables, 98 newly diagnosed type 2 diabetic patients and 98 age- and sex-matched healthy controls were recruited from individuals undergoing routine health examinations at Tongji Hospital in Wuhan (Hubei, People's Republic of China) between December 2012 and May 2017. Oral glucose tolerance test (OGTT) was performed in all of the included participants. They had no history of drug intake including antihypertensive agent or lipid-lowering medication. Subjects with cardiovascular diseases, gestational diabetes, chronic renal failure, and active liver cirrhosis were excluded from the study. All participants did not received antidiabetic medication or insulin therapy, therefore, did not have glycemic control. Type 2 diabetes was diagnosed based on the American Diabetes Association guideline [17]. Type 1 diabetic patients were carefully excluded in our study on clinical grounds, from a review of medical records, on the basis of fasting C-peptide levels, and from negative islet-related autoantibodies. This study was approved by the institutional review board of Tongji Hospital and was carried out in accordance with the principles expressed in the Declaration of Helsinki. An informed written consent was obtained from all participants before their enrolment in the study.

### 2.2. Anthropometric and Biochemical Measurements

Blood samples were collected after an overnight fast in the morning in order to avoid potential confounding influences. Serum and plasma were stored in aliquots without preservatives at −80°C. Serum and plasma parameters were determined at the Department of Medical and Chemical Laboratory Diagnostics of Tongji Hospital according to routine procedures. The homeostasis model assessment of insulin secretion (HOMA-IS) and the homeostasis model assessment of insulin resistance (HOMA-IR) were calculated from fasting insulin and glucose levels with the following equations: HOMA‐IS = [20 × fasting insulin(IU/ml)]/[fasting glucose(mmol/l)–3.5] and HOMA‐IR = fasting glucose(mmol/l) × fasting insulin(mU/l)/22.5.Circulating cathepsin D concentrations were determined using commercially available enzyme-linked immunosorbent assays (ELISAs; EK0672, Boster, Wuhan, China) according to the manufacturer's instructions. All samples were analyzed in duplicate. Inter- and intra-assay coefficient of variation was <10%.

### 2.3. Standard Echocardiographic Measurements

A standard echocardiographic examination was performed in all type 2 diabetic patients (*GE Vingmed Vivid 7 or Vivid 9*, Horten, Norway). Left ventricular end-diastolic dimension (LVEDD), left ventricular end-systolic dimension (LVESD), and fractional shortening (FS) were measured using M-mode in the parasternal LV long axis view. Left ventricular biplane Simpson method ejection fraction (EF) was measured in apical 4- and 2-chamber views. The parameters of LV diastolic function were measured by recording transmitral flow velocity using Doppler echocardiography. The peak velocities of early (E velocity) and late (A velocity) transmitral flow velocities were measured, and the E/A ratio was calculated. The Tei index, which reflects both systolic and diastolic function, was obtained from tissue Doppler as previously published [18]. The tissue Doppler-derived Tei index was calculated: (MCOT-ET)/ET, where MCOT is the time interval representing the mitral valve closure-to-opening time and ET is the ejection time of the left ventricular outflow. The Tei index values from 3 cardiac cycles were averaged.

### 2.4. Statistical Analysis

Data are presented as means ± standard deviation (SD), median (25th and 75th percentiles) for continuous variables, or as percentage for categorical variables. The distribution of quantifiable variables was tested for normality using a one-sample Kolmogorov–Smirnov test. Comparisons of quantitative variables among groups were performed by one way ANOVA with post hoc Tukey's test. However, data that were not normally distributed were logarithmically transformed before analysis. Categorical variables were examined by Chi-squared test. Correlation analysis between continuous variables was performed by Pearson's analysis, and multiple testing was adjusted using Bonferroni correction. Statistical and association analyses were performed using SPSS 15.0 (SPSS Inc., Chicago, Illinois, USA). All tests were two-sided, and *P* values less than 0.05 were considered statistically significant.

## 3. Results

The clinical characteristics of the study population for both nondiabetic subjects and newly diagnosed type 2 diabetic patients are shown in Table 1. The sample cohort contains 98 type 2 diabetes cases and 98 ethnically and geographically matched controls. Because subjects were matched for age and sex, both parameters were similar between the two subgroups. Newly diagnosed type 2 diabetic patients had significantly higher levels of cathepsin D in BMI, systolic blood pressure (BP), diastolic BP, total cholesterol, LDL cholesterol, and triglyceride than those in control subjects. HbA1c, fasting glucose, and HOMA-IR were higher in type 2 diabetic patients than in control subjects as expected. There were no significant differences in HDL cholesterol, the frequency of smokers, and HOMA-IS between two groups. Thus, the islet beta cell secretory capacity was apparently preserved in newly diagnosed type 2 diabetic patients.

Of note, compared with the control group, circulating cathepsin D concentrations in newly diagnosed type 2 diabetic patient group were significantly increased (median level: 227 ng/ml versus 174 ng/ml, *P* < 0.01) (Table 1). Next, we studied correlations between cathepsin D levels and clinical variables. As shown in Table 2, circulating cathepsin D levels were correlated positively with BMI, triglyceride, HbA1c, fasting glucose, and HOMA-IR in all subjects (all *P* < 0.05). However, most of these associations disappeared after stratification analysis. In newly diagnosed type 2 diabetic patients, a highly significant correlation was found between cathepsin D levels and HOMA-IR (*r* = 0.25, *P* = 0.01). Furthermore, cathepsin D levels tended to marginally correlate positively with fasting glucose in the case group (*r* = 0.19, *P* = 0.06). In contrast, no significant correlation was found between cathepsin D levels and clinical parameters in the healthy control group (all *P* > 0.05).

**Figure 1:**
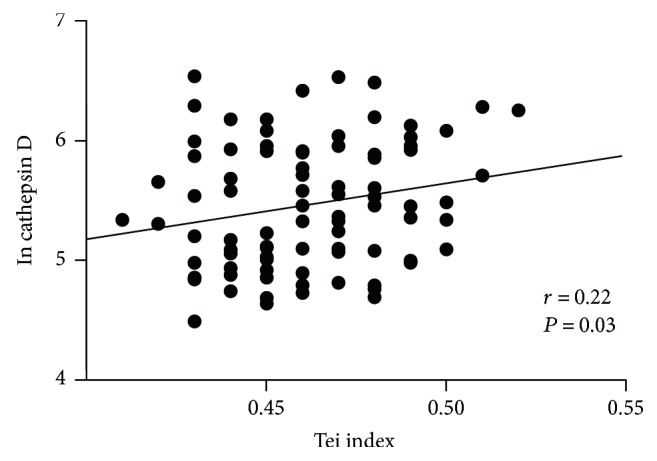
Correlations of circulating cathepsin D levels with the Tei index in type 2 diabetic patients.

The echocardiographic parameters of type 2 diabetic patients are summarized in Table 3. The mean E/A ratio is 1.17 ± 0.04. The mean FS (%) is 31.6 ± 2.1. The mean EF (%) is 63.1 ± 2.2. The mean LVESD (mm) is 30.9 ± 1.3. The mean LVEDD (mm) is 45.3 ± 1.9. The mean Tei index is 0.46 ± 0.02. Linear correlation analysis was performed to examine the relationship between the cathepsin D level and echocardiographic parameters. However, only the Tei index showed a weak correlation with cathepsin D level (*r* = 0.22, *P* = 0.03) (Figure 1). Other echocardiographic parameters did not correlate with cathepsin D level (all *P* > 0.05).

## 4. Discussion

The present study provides the first evidence that increased levels of circulating cathepsin D are associated with newly diagnosed and untreated type 2 diabetic patients. Furthermore, our study not only suggested the findings of cathepsin D associated with metabolic variables but also provided several novel findings. Our results showed that circulating cathepsin D concentrations were positively correlated with indexes of insulin resistance. Especially among newly diagnosed type 2 diabetic patients, cathepsin D were strongly correlated with insulin resistance. In agreement with previous finding [12], our data indicate that circulating cathepsin D levels associate positively with triglyceride levels in all participants.

Previously, data have shown that cathepsin D is a critical contributor to many steps of tumor progression, including the stimulation of cancer cell proliferation, the inhibition of tumor apoptosis, and growth of micrometastasis [19]. Recent study has identified that increased cathepsin D protein expression is a biomarker for bone malignancies [20]. Several studies have found that cathepsin D affects various different steps in tumor progression and metastasis, including fibroblast outgrowth and tumor angiogenesis [21]. It is noteworthy that most of the current literature on cathepsin D and cancer consider the enzymatically inactive precurs, procathepsin D, to be the relevant molecule. However, the present study used antibodies binding to both procathepsin and mature cathepsin, this option needs to be investigated in the future. On the other hand, multiple lines of evidence have demonstrated that cathepsin D participates in the pathogenesis of atherosclerosis. Moreover, enhanced cathepsin D activation has been found in patients with cardiovascular events [16, 22]. It has also been reported that altered expression of cathepsin D levels has been linked to Alzheimer's disease [14, 23], indicating the possible role of cathepsin D in regulating tau degradation. Taken together, these results suggest that cathepsin D not only represents a cancer biomarker but also implicated in the pathogenesis of tau degradation and atherosclerosis.

Accumulating evidence demonstrated that obesity, low-grade chronic inflammation, and increased production of proinflammatory cytokines play crucial roles in the development of insulin resistance [24]. It has been reported that key cathepsin, cathepsin D, is activated at the early stages of weight gain and obesity [25]. Notably, cathepsin D was suggested as a potential mediator that contributes to obesity, chronic inflammation, and insulin resistance through influenced detoxification of advanced glycation end products and proapoptotic protein activation [26]. Importantly, recent data show that circulating levels of cathepsin D are increased in individuals with insulin resistance in nondiabetic community residents [7]. It is not clear whether oral hypoglycemic agents will affect serum cathepsin D level or not. Therefore, only newly diagnosed type 2 diabetic patients were included in the current study. The observed strong association between IR and cathepsin D in the present study may be resulting from lipotoxicity and inflammation in insulin-resistant states. Although no evidence of a causal effect of IR on cathepsin D was found by Mendelian randomization analysis, future studies are needed to investigate the implication of cathepsin D in insulin resistance and diabetes. In the current study, our data are the first to report that the increased levels of circulating cathepsin D are associated with newly diagnosed and untreated type 2 diabetic patients. In accordance with previous evidence, our study has confirmed that cathepsin D levels were positively associated with insulin resistance. Similarly, previous study showed the positive correlation between serum concentration of cathepsin D and carotid intima-media thickness [12]. Consistently, cathepsin D levels are reported to negatively correlate with endothelial dysfunction in chronic kidney disease [27]. Alternatively, reduced level of cathepsin D was also observed in patients with preeclampsia in a Korean population [28]. Moreover, circulating cathepsin D concentrations are reported to associate with human aging [29]. These findings suggest that cathepsin D has emerged as a key molecular implicated in various diseases. Currently, the precise mechanisms underlying elevated circulating cathepsin D levels in newly diagnosed type 2 diabetic patients remain uncertain. Thus, future studies will be needed to clarify the role of cathepsin D in the pathogenesis of insulin resistance.

Increasing evidences point to a strong link between cathepsin D and cholesterol-mediated inflammation [30]. Likewise, cathepsin D has been suggested to participate in the apoptosis of macrophage foam cells, a determinant of plaque instability [8]. In line with this, previous study showed that cathepsin D could be a marker for early diagnosis and target for treatment of cardiovascular diseases in patients with chronic kidney disease [27]. The Malmö Diet and Cancer Cardiovascular Cohort study revealed that high plasma level of cathepsin D was associated with increased risk of future coronary events during a mean follow-up time of 14.0 ± 4.3 years [31]. Recently, high circulatory levels of cathepsin D were shown to be inversely associated with deterioration in cardiac function in patients with acute myocardial infarction [16]. Interestingly, our data also suggested that the level of cathepsin D was inversely associated with cardiac dysfunction as assessed by Tei index. The potential of the Tei index, myocardial performance index, as a clinically meaningful diagnostic and prognostic marker has been demonstrated in previous studies investigating heart failure [32, 33]. It is well known that the Tei index has the unique merit that it does not depend on ventricular geometry and image quality. Although the Tei index is an easy-to-obtain parameter, it reflects combined systolic and diastolic myocardial performance. Accumulating evidence suggested that the prevalence of advanced cardiac dysfunction was remarkably high in type 2 diabetes. Several studies showed that the Tei index was impaired in type 2 diabetes and correlated with plasma B-type natriuretic peptide levels [34]. Previous data demonstrated the potential clinical usefulness and the merit of the Tei index in assessing advanced cardiac dysfunction. Emerging evidences reveal that diabetes is associated with functional and structural abnormalities of the myocardium, leading to diabetic cardiomyopathy. Consistently, the most striking result of the present analysis was that the cathepsin D level was associated with the Tei index. This might have important pathophysiological implications regarding diabetic cardiomyopathy. However, the mechanism underlying these processes still remains largely unknown. Furthermore, these findings need to be confirmed in future studies with larger sample sizes.

In conclusion, we provide the first evidence that increased level of cathepsin D serves as a novel determinant of type 2 diabetes and measurement of circulating levels may be helpful for assessment of diabetes risk. Moreover, the present study shows that circulating cathepsin D concentrations not only positively correlated with indexes of insulin resistance but also correlated with myocardial performance index. Therefore, cathepsin D may have clinical significance for the early identification of individuals at high risk of insulin resistance and cardiac dysfunction. Further studies are required to elucidate the role of cathepsin D in the pathogenesis of insulin resistance and cardiac dysfunction.

## Figures and Tables

**Table 1:** Clinical characteristics of control subjects and newly diagnosed type 2 diabetic patients.

Characteristics	Control (*n* = 98)	Case (*n* = 98)	*P* value
Male (%)	56 (57.1)	56 (57.1)	—
Age (years)	51.8 ± 7.1	52.1 ± 7.4	0.81
BMI (kg/m^2^)	22.5 ± 2.7	24.6 ± 2.6	<0.01
Smoking (%)	24 (24.5)	33 (33.7)	0.21
Systolic BP (mmHg)	123 ± 11	129 ± 13	<0.01
Diastolic BP (mmHg)	79 ± 7	83 ± 8	<0.01
Total cholesterol (mmol/l)	4.6 ± 0.9	5.0 ± 1.2	0.01
Triglyceride (mmol/l)	1.5 ± 0.9	1.7 ± 0.7	0.04
LDL cholesterol (mmol/l)	2.6 ± 0.7	2.9 ± 0.6	<0.01
HDL cholesterol (mmol/l)	1.33 ± 0.30	1.29 ± 0.32	0.40
HbA_1c_ (%)	5.7 ± 0.3	6.5 ± 0.4	<0.01
Fasting glucose (mmol/l)	5.8 ± 0.4	8.2 ± 1.1	<0.01
HOMA-IR	1.8 ± 0.9	5.1 ± 1.8	<0.01
HOMA-IS	58.8 ± 25.4	63.1 ± 26.9	0.25
Cathepsin D (ng/ml)	174 (138, 240)	227 (157, 362)	<0.01

Data are means ± SD, *n* (%), and median (25th and 75th percentiles). BMI: body mass index; BP: blood pressure; HbA_1c_: hemoglobin A1c; LDL: low-density lipoprotein; HDL: high-density lipoprotein; HOMA-IR: homeostasis model assessment of insulin resistance; HOMA-IS: homeostasis model assessment of insulin secretion.

**Table 2:** Correlation between circulating cathepsin D levels and clinical parameters.

Variables	All subjects (*n* = 196)		Control (*n* = 98)		Case (*n* = 98)	
	Coefficient	*P* value	Coefficient	*P* value	Coefficient	*P* value
Age (years)	−0.01	0.87	−0.01	0.90	−0.02	0.85
BMI (kg/m^2^)	0.19	0.01	0.10	0.31	0.12	0.23
Systolic BP (mmHg)	0.08	0.27	0.05	0.60	0.01	0.97
Diastolic BP (mmHg)	0.04	0.61	−0.03	0.74	−0.01	0.97
Total cholesterol (mmol/l)	0.07	0.33	0.04	0.69	0.02	0.86
Triglyceride (mmol/l)	0.18	0.01	0.17	0.10	0.15	0.15
LDL cholesterol (mmol/l)	0.04	0.60	0.12	0.25	−0.15	0.15
HDL cholesterol (mmol/l)	−0.02	0.74	0.06	0.56	−0.07	0.50
HbA_1c_ (%)	0.16	0.02	−0.04	0.69	0.02	0.86
Fasting glucose (mmol/l)	0.26	<0.01	−0.01	0.99	0.19	0.06
HOMA-IR	0.29	<0.01	0.02	0.33	0.25	0.01
HOMA-IS	0.09	0.22	0.13	0.19	0.02	0.84

Circulating cathepsin D levels were log-transformed variable. BMI: body mass index; BP: blood pressure; HbA_1c_: hemoglobin A1c; LDL: low-density lipoprotein; HDL: high-density lipoprotein; HOMA-IR: homeostasis model assessment of insulin resistance; HOMA-IS: homeostasis model assessment of insulin secretion.

**Table 3:** Correlation between circulating cathepsin D levels and echocardiographic data in type 2 diabetic patients.

Variables	Type 2 diabetes (*n* = 98)	Coefficient	*P* value
E/A ratio	1.17 ± 0.04	0.02	0.83
FS (%)	31.6 ± 2.1	0.10	0.29
EF (%)	63.1 ± 2.2	0.12	0.25
LVESD (mm)	30.9 ± 1.3	0.01	0.96
LVEDD (mm)	45.3 ± 1.9	0.08	0.42
Tei index	0.46 ± 0.02	0.22	0.03

Circulating cathepsin D levels were log-transformed variable. Data are means ± SD. FS: fraction shortening; EF: ejection fraction; LVESD: left ventricular end-systolic diameter; LVEDD: left ventricular end-diastolic diameter.
